# Does Hypohydration Really Impair Endurance Performance? Methodological Considerations for Interpreting Hydration Research

**DOI:** 10.1007/s40279-019-01188-5

**Published:** 2019-11-06

**Authors:** Lewis J. James, Mark P. Funnell, Ruth M. James, Stephen A. Mears

**Affiliations:** 1 School of Sport, Exercise and Health Sciences, National Centre for Sport and Exercise Medicine, Loughborough University, Loughborough, Leicestershire, LE11 3TU UK; 2 Sport, Health and Performance Enhancement Research Centre, School of Science and Technology, Nottingham Trent University, Nottingham, UK

## Abstract

The impact of alterations in hydration status on human physiology and performance responses during exercise is one of the oldest research topics in sport and exercise nutrition. This body of work has mainly focussed on the impact of reduced body water stores (i.e. hypohydration) on these outcomes, on the whole demonstrating that hypohydration impairs endurance performance, likely via detrimental effects on a number of physiological functions. However, an important consideration, that has received little attention, is the methods that have traditionally been used to investigate how hypohydration affects exercise outcomes, as those used may confound the results of many studies. There are two main methodological limitations in much of the published literature that perhaps make the results of studies investigating performance outcomes difficult to interpret. First, subjects involved in studies are generally not blinded to the intervention taking place (i.e. they know what their hydration status is), which may introduce expectancy effects. Second, most of the methods used to induce hypohydration are both uncomfortable and unfamiliar to the subjects, meaning that alterations in performance may be caused by this discomfort, rather than hypohydration per se. This review discusses these methodological considerations and provides an overview of the small body of recent work that has attempted to correct some of these methodological issues. On balance, these recent blinded hydration studies suggest hypohydration equivalent to 2–3% body mass decreases endurance cycling performance in the heat, at least when no/little fluid is ingested.

## Key Points

**Table:** 

Previous work that has investigated the effect of hypohydration on endurance exercise performance may be confounded by the lack of study blinding or the contrived and uncomfortable methods used to induce hypohydration
Recent work has attempted to correct these issues by using more robust methods, although this work is only in its infancy and, at present, limited to endurance cycling performance
On balance, these studies suggest hypohydration equivalent to 2–3% body mass impairs endurance exercise performance in the heat, at least when no/little fluid is ingested

## Introduction

The effect of dehydration/hypohydration on exercise performance and related responses has been extensively researched over the past century. Despite the long history of research in the area, there is still significant controversy, and the effects of dehydration/hypohydration are still much debated. Some of this debate pertains to the methodologies used to study how changes in hydration influence exercise performance, but this topic, specifically, has not been the focus of any previous review. This narrative review discusses the methods used in hydration research, and their possible implications on performance outcomes, with a specific focus on endurance exercise. In particular, we focus on recent contemporary work using novel methods to more robustly explore the effects of hypohydration on endurance exercise performance. Whilst we provide some background detail, this is more used to set the scene, rather than providing a thorough review. Therefore, interested readers are directed to recent review articles on physiological/performance effects of dehydration/hypohydration [1–5] and guidelines for drinking during exercise [6–9], as well as interesting recent articles documenting the contrasting views of researchers in the field [10–12].

## Water Balance

### Setting the Scene

Water is the most abundant molecule in the human body, making up 60–70% of an adult athlete’s body mass. Despite its abundance, body water is tightly regulated, with normal daily variation considered to be a change of ≤ 1% of body mass [1]. Euhydration refers to a normal state of body water, with deviations from this norm producing compensatory responses that act mainly through altering renal water concentration and thirst sensation [1]. A sustained increase in body water, although often transient, is referred to as hyperhydration, whilst hypohydration refers to a sustained decrease in body water [13]. The term dehydration refers to the process of losing water, rather than a state of low body water, but because changes in body water evoked by exercise are typically short lived, the terms dehydration and hypohydration are often used interchangeably in this context. In this review, we use hypohydration to refer to a body water loss and to situations where an exercise performance test commences with a body water loss and dehydration to refer to the process of losing water or when body water losses accrue during exercise performance.

On a daily basis, water losses through urine, sweat, respiration, faeces and the skin are offset against gains through food and drink, as well as metabolic water formation. In sedentary humans, gains to the body water pool (except metabolic water formation) are episodic in nature, mainly occurring in/around discrete mealtimes [14]. In contrast, losses from the body water pool (except perhaps drink-induced diuresis) occur continuously. These patterns of water gain and loss mean that euhydration is not a single point but oscillates over the day, representing a range of total body water values [13]. Exercise can produce sweat rates > 3–4 L/h in some athletes, although 1–2 L/h is more typical [15], hence sweat lost during exercise can present a significant challenge to body water homeostasis, particularly when exercise training is prolonged or performed in hot/humid environments.

### Endurance Exercise and Water Balance

Typically, fluid ingested during exercise is insufficient to keep pace with sweat losses, meaning hypohydration may accrue during prolonged exercise [15–17]. Furthermore, when training frequency is high, recovery periods can be short, making complete rehydration between sessions problematic [18]. Consistent with this, elevated pre-exercise urine concentration, indicative of hypohydration, or at least an attempt by the kidneys to conserve water, has been reported [19]. For athletes, a body water deficit could be present at the start of exercise and/or might develop during exercise.

Whilst pre-exercise hypohydration might present in training, tapering, coupled with targeted nutrition strategies that precede competitive events, make it far less likely that athletes will commence competition hypohydrated. However, exercise-induced dehydration is still a likely outcome [15, 16]. In this setting, the degree of hypohydration accrued during exercise will be the product of the net rate of water loss and the exercise duration and may produce a substantial body water loss towards the end of exercise. Given that performance in the latter stages of competition, at least in endurance sports, is often what separates athletes, any negative effects of exercise-induced dehydration could have significant implications for competitive success.

If sufficiently large, hypohydration can result in heat syncope, brought about by venous pooling and a reduction in brain blood flow [20] or heat exhaustion [21]. Clearly, these symptoms are not compatible with optimal performance, and consequently the pertinent question is actually *when*, not *if,* hypohydration will impair performance. Typically, hypohydration experienced by athletes is equivalent to ~ 1 to 5% body mass [16, 22–25] and might have consequences for performance [26–44]. This is not a new question (see Pitts et al. [45]), but there is now a huge body of work in this area, mainly showing that hypohydration of ≥ 2% body mass impairs endurance performance [1, 3, 17], particularly in hot environments [4]. Despite the substantial evidence available, whether hypohydration impairs performance is still hotly debated [6, 10, 46]. Most of this debate centres on how the results of studies are incorporated into drinking guidelines, with some arguing fluid intake during exercise should be planned, whilst others argue simply drinking to thirst is optimal (see Holland et al. [3], Cotter et al. [6] and Kenefick [9] for detailed reviews).

When considering water losses during exercise, it is important to note that body mass loss is only a proxy for water loss, with some of the mass loss derived from stores other than water (i.e. substrate) [47]. Whilst non-water-derived mass loss might be substantial during ultra-endurance exercise [47], it is likely relatively small during 2–3 h of exercise (perhaps ~ 0.5% body mass). However, these other losses mean that maintenance of euhydration does not mean maintenance of body mass during endurance exercise.

### Hypohydration and Endurance Performance

Sweat secreted during exercise is hypotonic relative to serum [15], with the exception of some genetic conditions (e.g. cystic fibrosis [48]). Therefore, exercise-induced sweating produces a proportionally greater loss of water than solute, which, combined with inadequate fluid intake during exercise, leads to a reduced plasma volume and consequent increase in extracellular osmolality [1]. This creates a concentration gradient resulting in water movement from the intracellular fluid to the extracellular fluid, meaning water losses are partitioned between the fluid spaces. The hypohydration produced in these settings is termed intracellular hypohydration or hypertonic hypovolemia [1]. Similar effects on body water are observed as a result of inadequate daily fluid intake [49, 50] or inadequate post-exercise rehydration [51]. Therefore, hypertonic hypovolemia is the prevalent form of hypohydration experienced by athletes in most exercise settings, except perhaps during altitude exposure [1].

The increased extracellular osmolality when hypohydration is produced by exercise is important for the coordinated regulatory response that leads to the replacement of body water losses. An increased extracellular osmolality of ~ 2% (i.e. ~ 6 mosmol/kg) stimulates arginine vasopressin (AVP) secretion, which decreases water loss by promoting renal water reabsorption, as well as stimulating thirst to prompt behavioural responses that facilitate water intake [1].

**Fig. 1:**
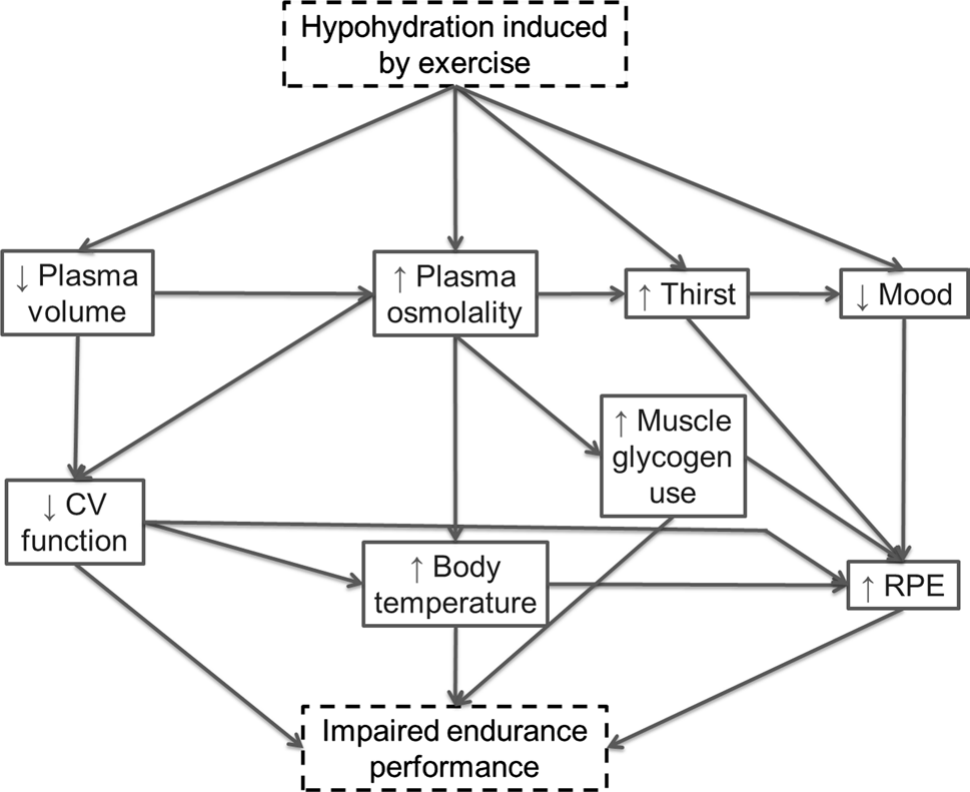
Basic flow diagram representing how exercise-induced dehydration might impair endurance exercise performance. *CV* cardiovascular, *RPE* rating of perceived exertion, ↑ increased, ↓ decreased

Hypohydration seems to impair endurance performance through a combination of mechanisms, principally driven by hypovolemia [4]. This hypovolemia and the resultant hyperosmolality precipitate a cascade of physiological and perceptual responses that seemingly act in concert to reduce endurance performance. From a physiological perspective, these responses include reductions in muscle [52] and cerebral [53] blood flow, increased body temperature [54], increased heart rate/cardiovascular strain [55] and increased muscle glycogenolysis [56], possibly limiting peak oxygen uptake [57]. From a perceptual standpoint, hypohydration stimulates thirst [58, 59] and lowers mood [60–62], producing a level of discomfort that may distract from the task at hand. Ultimately, these physiological and perceptual responses likely act in combination to increase perception of effort at a given intensity, thereby compromising performance [63, 64] (Fig. 1).

Hypohydration of ≥ 2% body mass has been shown to impair endurance performance and capacity across a range of exercise modalities and durations [26–44]. There appears to be an interaction between hypohydration and ambient conditions, whereby the negative performance effects are exacerbated in hotter conditions [30, 34]. Hot skin, caused by a hot environment, increases peripheral vasodilation and competes with the working muscle for blood flow demands, ultimately increasing cardiovascular strain [4]. Not all studies report that hypohydration impairs performance [65–69]. However, only studies where exercise is short in duration (~ 1 h) and in which a high rate of fluid intake is required to maintain euhydration report attempting to maintain euhydration may negatively affect performance, likely owing to issues relating to gastrointestinal comfort [2, 70, 71]. 

Some argue that thirst, rather than hypohydration, limits performance [6, 10, 46]. It certainly seems intuitive that thirst would be one of several factors influencing performance (Fig. 1), particularly given the importance of water acquisition in sustaining life. This has produced two schools of thought related to guidelines for fluid intake during exercise, namely *‘planned drinking’* and *‘thirst*-*driven drinking’* (for reviews see Cotter et al. [6] and Kenefick [9]). Whilst much important debate has focused on these guidelines and their potential consequences, we feel some important methodological issues fundamental to laying the foundations of hydration performance research have largely been ignored. Therefore, whilst discussions related to drinking strategies are important, this review focusses on unpicking the possible effects of the methodology used in hydration research, and attempts to establish if there is good evidence to suggest that hypohydration impairs performance.

## Methodology

### Methodological Limitations in Hydration Research

Whilst there is a clear mechanistic basis for how hypohydration might impair endurance performance, methodological limitations perhaps make it difficult to ascertain the true effects at levels of hypohydration typically experienced by athletes. There are two main limitations of the evidence base in this area. First, until recently, no performance study had blinded subjects to their hydration status. Second, many of the methods used to induce hypohydration are both uncomfortable and unfamiliar to subjects. These limitations in the present literature make it difficult to draw robust conclusions.

### Blinding Changes in Hydration

In sport and exercise nutrition research, studies examining the performance effects of a nutrition strategy (e.g. carbohydrate intake or dietary supplements) use blinded experimental designs to remove any potential associated placebo/nocebo effects, but this is not the case with hydration research. Previous research clearly demonstrates placebo effects are evident for carbohydrate [72], caffeine [73], sodium bicarbonate [74] or breakfast [75] consumption before exercise. Indeed, it seems unlikely that any reputable scientific journal would accept for publication a study examining the performance effects of a known ergogenic supplement if the subjects were not blinded to their treatments. However, this is exactly the case for studies examining differences in hydration, as the methods used to induce hypohydration (e.g. fluid restriction before/during exercise, heat exposure, diuretic administration) are overt, meaning subjects know which trials they are performing. Because athletes believe that hypohydration, at least significant hypohydration, impairs performance [76], the overtness of the methods used to elicit hypohydration might entirely explain the results observed. This is potentially a serious problem, requiring attention before we can establish the true performance effects of hypohydration. To date, studies have used two different methods to achieve this blinding: intravenous delivery of fluids [44, 67, 68], or delivery of fluid to the stomach through a gastric feeding tube [41–43].

In the first study of its type, Wall et al. [67] dehydrated trained cyclists by 3% body mass through exercise, followed by intravenous rehydration with approximately isotonic fluid in the 2-hour post-exercise, producing euhydration or hypohydration of ~ 2% and ~ 3% body mass at the start of a 25-km cycling time trial in 33 °C. Performance was similar between trials. Using a similar study design, Cheung et al. [68] produced euhydration or hypohydration (~ 2.1% body mass) via an intravenous infusion of isotonic saline during a 90-min standardised preload in trained cyclists. Subjects then completed a 20-km cycling time trial in 35 °C. Subjects performed two euhydrated and two dehydrated trials, with ad libitum mouth rinsing of water in one trial of each to remove sensations of thirst/dry mouth. Again, time trial performance was similar between trials, suggesting that neither hypohydration nor thirst influences performance. These interesting studies successfully blinded subjects to their hydration status and have potentially important implications. They suggest that when an individual’s knowledge of their hydration status is removed, hypohydration of 2–3% body mass does not impair exercise performance, meaning previous studies were potentially confounded by the lack of blinding. Interestingly, both these studies reported higher rectal temperature with hypohydration towards the end of their time trials, which in some exercise settings, might impair performance capabilities [77].

**Fig. 2:**
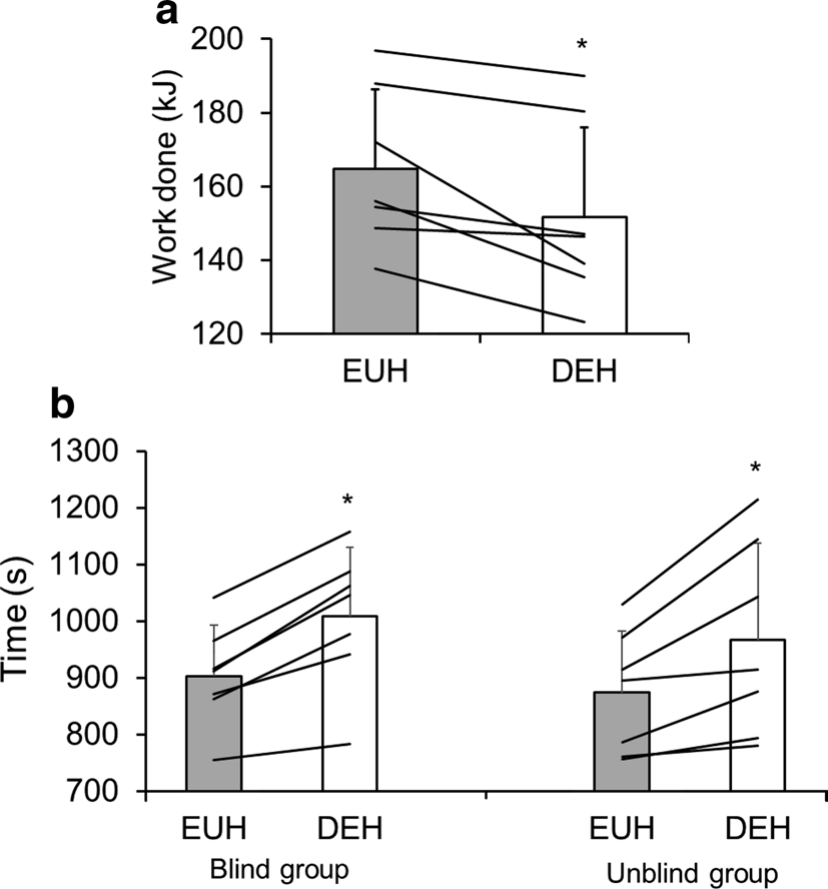
**a** Total work done (kJ) in a 15-min cycling performance test in blinded euhydrated (EUH) and dehydrated (DEH) trials (adapted with permission from James et al. [41]). **b** Time to complete a set amount of work (s) in blinded EUH and DEH trials (adapted with permission from Funnell et al. [43]). Bars are mean ± standard deviation, lines are individual subject data. *Indicates DEH performance significantly worse than EUH

Our group has used a different approach to blind subjects, choosing to deliver water (or not, as the case may be) directly to the stomach via a gastric feeding tube inserted orally [41] or nasally [43]. In our first study [41], active but not specifically cycling-trained subjects completed eight blocks of 15 min cycling, separated by 5 min rest, in 34 °C. Water infused into the stomach was manipulated to either maintain euhydration or produce hypohydration (~ 2.4% body mass) by the end of the preload. Additionally, a small volume of water (~ 15 mL) was orally ingested every 10 min of the preload in both trials. Subjects then performed a performance test, where they had to complete as much work as possible in 15 min. To remove expectation effects related to hydration and assist with the blinding, subjects were told that drink composition was being manipulated. In contrast to our hypothesis and the results of previous studies [67, 68], 8% less work was completed in the hypohydrated trial (Fig. 2a). Subsequently, we used the same methods to explore the effects of blinded hypohydration in trained cyclists, except the 2-hour cycling preload was continuous rather than intermittent [43]. This time we recruited two pair-matched groups of trained cyclists, with both groups completing trials in 31 °C. Water intake was manipulated to either maintain euhydration or produce hypohydration (~ 3% body mass) at the end of the preload. Subjects then completed a time trial, where they performed a set amount of work (estimated to last ~ 15 min) as quickly as possible [78]. One group (blinded group) had water delivered through a nasogastric feeding tube with a similar cover story and oral water intake to our previous study [41], whilst in the other group (unblinded group) all water was provided orally in both trials. We hypothesised that hypohydration would impair performance in both groups, but that the impairment would be attenuated in the blinded group. In contrast to this hypothesis, we observed that the performance effects were remarkably similar between groups with performance decrements of 11% (blinded group) and 10% (unblinded group) caused by the hypohydration (Fig. 2b).

It is important to note that careful consideration needs to be given to the finer points of the methods used in these gastric infusion studies to ensure adequacy of blinding. First, the gastric tube must be fixed in position, by application of tape on the cheek, behind the ear, and onto the centre of the upper back, and dummy infusions must occur in dehydrated trials. Fluid infused must be maintained at body temperature (~ 37 °C), as too much deviation from this value will mean fluid is detected when it travels down the tube into the stomach. Fluid should be infused in small volumes every few minutes, as infusions of large volumes will increase gastric distension and may be detected by subjects. Finally, we recommend a clear, coherent and plausible cover story is used to remove any pre-conceived ideas about the effects of hypohydration and, if thirst might respond differently between trials, prevent subjects determining which trial they are completing.

The inconsistent findings between these blinded hydration studies [41, 43, 67, 68] suggest that further research is required before a firm conclusion can be made. However, perhaps differences in the methods used might also explain the results, as studies using intravenous rehydration report hypohydration does not impair performance [67, 68], whilst studies using intragastric rehydration do [41, 43]. These apparent differences might be caused by the divergent effects on physiology and perception produced by the different techniques.

Manipulating hydration status via intragastric water delivery produces differences in serum osmolality between euhydrated and hypohydrated trials [41, 43], caused by hypovolemia with hypohydration. Because serum osmolality is key for coordinating physiological fluid balance responses to hypohydration induced by exercise [1], it is likely that replicating typical responses is important in blinded hydration studies. The use of approximately isotonic saline in studies manipulating hydration status through intravenous rehydration [67, 68] means the hypertonicity produced is present (and similar) in both hypohydrated and euhydrated trials. As such, internal homeostatic signals indicative of hypohydration (i.e. an increased AVP concentration and renal water conservation, as well as intracellular dehydration) were likely activated in both trials. Thus, in these studies [67, 68], the internal physiological milieu would be consistent with, and likely sensed as, hypohydration, irrespective of body water. In contrast, euhydration/hypohydration induced by intragastric rehydration produces physiological responses consistent with those typically reported in unblinded studies (i.e. decreased plasma volume, increased serum osmolality, increased AVP concentration). Therefore, whilst body water might be manipulated by intravenous rehydration, if internal physiological signals still indicate hypohydration, this might feed into the control of self-regulated exercise intensity and performance [63, 64].

Additionally, perceptual responses related to fluid balance (e.g. thirst) might play a key role in how hypohydration impairs endurance performance [6, 8, 46], particularly as oral fluid ingestion may be important for fluid balance regulation [79]. In a hypohydrated state (~ 2.8% body mass loss) induced by exercise, ingestion of water followed by extraction from the stomach suppresses thirst and AVP (at least partially) within minutes, despite no recovery of plasma volume or osmolality either immediately or in the subsequent 80 min [79]. Additionally, Arnaoutis et al. [80] reported that exercise capacity in 31 °C, when hypohydrated (~ 1.9% body mass loss induced by exercise), was increased with ingestion of, but not mouth rinsing with (25 mL in both situations) water, when compared with a no water trial. The exercise capacity test lasted ~ 20 min, thus the ~ 100 mL ingested in that trial was not meaningful for fluid balance. This and other work [58] demonstrate that mouth rinsing water alone confers no performance benefit, suggesting fluid must be consumed to influence performance. Casa et al. [81] reported a strong trend (*p *= 0.07) for oral rehydration to increase exercise capacity compared with intravenous rehydration in recovery from hypohydration (4% body mass). Interestingly, oral rehydration reduced rectal and skin temperatures during exercise, as well as reducing other relevant variables (blood lactate/glucose concentrations and respiration rate) compared with intravenous rehydration. This suggests that oral fluid intake might also be important for some of the physiological effects associated with euhydration.

Taken together, the limited evidence available suggests that the swallowing of fluid might be an important factor involved in both regulatory and performance responses to hypohydration during exercise. Some have speculated that this response is possibly related to activation of oropharyngeal receptors [79, 80]. However, it is difficult to separate effects evoked by oropharyngeal responses from those evoked by gastric responses because fluid ingested is received by the stomach. Thus, it is difficult to discern if the swallowing of fluid per se, or the delivery of fluid to the stomach/gastrointestinal tract, controls these effects. Either way, as no fluid was swallowed in the studies of Wall et al. [67] and Cheung et al. [68], this might explain why hypohydration did not affect performance. In contrast, our studies, using intragastric rehydration combined with some oral rehydration (~ 15 mL every 5 min in euhydrated and hypohydrated trials), would have activated receptors present in oropharyngeal and gastric regions, possibly explaining the performance responses observed. Adams et al. [42] also used intragastric rehydration to produce blinded euhydration or hypohydration (~ 2.2% body mass) at the end of 2 h of exercise in 35 °C. Additionally, 25 mL water was ingested every 5 min of the 2 h to suppress thirst. Thirst was similar between trials, whilst the mean work rate was ~ 6% lower in the hypohydration trial, suggesting hypohydration can impair performance independent of thirst. It is important to note that these results [42] should not be interpreted as thirst not playing a role in hypohydration-induced impairments of performance, but rather that the effects are not fully mediated by thirst.

The notion that oropharyngeal/gastrointestinal stimulation following drinking might be important for performance is supported by the results of another recent blinded study [44]. Intravenous rehydration was used to maintain euhydration or induce hypohydration (~ 1.5% body mass) with water ingested in both trials (25 mL every 5 min). In contrast to previous studies using intravenous rehydration for blinding [67, 68], hypohydration impaired endurance performance. However, it must be noted that the plasma volume expansion produced by saline infusion in the euhydrated trial could also explain the results, as pre-exercise plasma volume expansion of the magnitude observed has previously been shown to enhance performance [82]. However, taken together, these studies might suggest that oral fluid intake is necessary to maximise performance responses to euhydration. Indeed, this is a theory that reconciles the discordant performance responses observed in blinded hydration studies to date.

The findings of Funnell et al. [43] are particularly important for interpreting previous work investigating the performance effects of hypohydration, as one might hypothesise (as we did) that knowledge of hypohydration might exaggerate any negative performance effects. Therefore, these results suggest that when hypohydration of ~ 3% body mass is present, impairments in endurance performance are not caused or exaggerated by a lack of study blinding. This suggests the conclusions of previous work, where hypohydration was ≥ 3% body mass, are unlikely to be confounded. However, it is possible the negative performance effects of previous unblinded studies, where hypohydration is < 3% body mass (including our own), may be inflated or explained by a placebo/nocebo effect. At lower levels of hypohydration (< 1–2%), confidence that the change in body water is outside typical euhydrated fluctuations is reduced [1]. Thus, it seems likely that the lower the level of hypohydration, the greater the chance that any associated negative performance effects are exaggerated or explained by placebo/nocebo effects.

It is important to note that the aforementioned blinded hydration studies do not necessarily provide evidence about the mechanisms by which hypohydration influences performance. What these studies do is to begin to build a strong foundation on which to understand if hypohydration, at a level commonly experienced in athletic settings, influences performance. On balance, our view is that the evidence to date strongly suggests that when sufficient hypohydration is present (possibly > 2% body mass), endurance cycling performance in the heat is compromised, at least when all typical physiological and perceptual symptoms are present.

### Uncomfortable and Unfamiliar Dehydration Methods

As well as being overt, the methods used to induce hypohydration in the scientific literature are often atypical of subjects’ normal behaviour and produce uncomfortable symptoms/side effects. For example, common techniques used to induce hypohydration include: prolonged passive fluid restriction [32, 35], exercise-induced dehydration combined with fluid restriction during or after exercise [27, 40] or diuretic drug administration [26]. Fluid restriction causes thirst, whilst diuretic use can cause polyuria, both of which are uncomfortable. These dehydration methods increase feelings of headache [60] and can increase sensations of pain during exercise [61], likely explaining the negative influence of hypohydration on mood [60–62], as well as the impairment of performance of vigilance-related tasks [82]. Thus, some of the effects of hypohydration on performance reported in the literature might actually be associated with this discomfort, rather than hypohydration per se. A related methodological consideration here is drinking during exercise at a rate below what an athlete would do if provided fluid ad libitum, which is outside the scope of this review and interested readers are directed to recent review articles [3, 6, 7, 9].

There is considerable inter-individual variation with regard to tolerance of hypohydration [10]. Data from competitive endurance events report that greater body mass loss is weakly associated with better race performance [84–88]. This has, by some, been misinterpreted as evidence that hypohydration produced during prolonged endurance exercise might enhance performance. Clearly, association does not prove causation, but it seems likely the direction of the relationship would be the opposite, such that faster racing precipitates increased body mass loss (i.e. hypohydration). Faster racing means reduced time available to drink, increased metabolic heat production and sweat rate [89], and possibly decreased gastric emptying of ingested fluids with effects on gastrointestinal comfort [90]. This was nicely demonstrated by Dion et al. [91], who reported, in a controlled laboratory experiment with ad libitum drinking, that faster racing lead to a greater sweat rate and hypohydration at the end of a half marathon, but did not alter drink ingestion. However, the finding that endurance athletes can finish [80–84] and even win races in world-class times [92] with as much as 10% body mass loss is intriguing. Because of issues related to fluid availability (e.g. drink station placement or difficulties with transporting fluids) or gastrointestinal comfort at higher exercise intensities [90], well-trained endurance athletes are likely to regularly perform hypohydrated in their normal training and/or competition [16, 22–25]. This may increase resilience to hypohydration-induced discomfort and reduce the impact of hypohydration on performance, closing the gap between euhydrated and hypohydrated performance. Therefore, some researchers [6, 39, 93, 94], us included, have postulated that repeated exposure to hypohydration might mitigate some of the negative performance effects.

There is, however, limited empirical evidence to draw on at this time. Merry et al. [93, 94] reported that training status alters the fluid balance regulatory [93] and physiological [94] responses to hypohydrated exercise. Despite this, the performance impairment with hypohydration was similar in trained and untrained subjects [94]. However, one of the untrained subjects performed 16% better in the hypohydrated trial compared with the euhydrated trial, which seems unlikely. Removal of this subject changed the interpretation of the data, such that having a higher aerobic fitness attenuated the performance impairment caused by hypohydration. However, whilst greater aerobic fitness might alter physiological responses to hypohydrated exercise, we hypothesise that familiarity with a hypohydration stimulus, rather than fitness, might attenuate performance impairments. Therefore, the methods used to induce pre-exercise hypohydration by Merry et al. [94] were possibly unfamiliar to both groups.

**Fig. 3:**
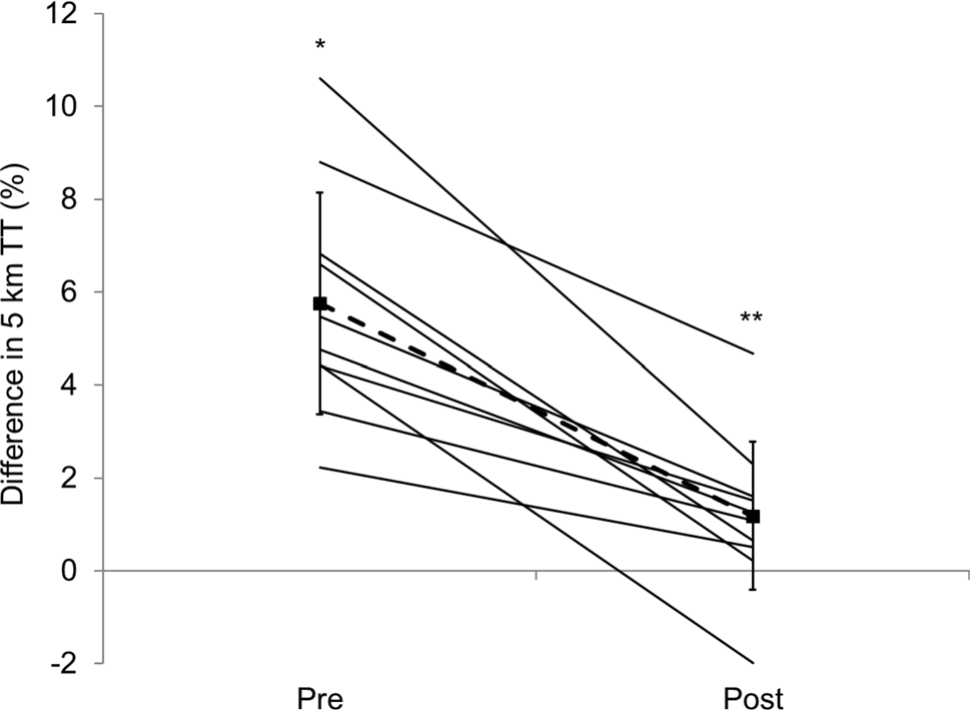
Percentage difference in 5-km running performance for hypohydration vs. euhydrated trials before and after four trials to familiarise subjects with the methods used to induce hypohydration. Points joined by a dashed line are mean ± standard deviation. Solid lines are individual subject data (adapted with permission from Fleming and James [39]). *TT* time trial, *indicates hypohydration performance significantly worse than euhydrated performance, **indicates performance impairment caused by hypohydration significantly reduced in post-familiarisation trials compared with pre-familiarisation trials

To date, the study by Fleming and James [39] is the only study to directly assess the effect of repeated familiarisation with hypohydration. In this study, active, but not endurance-trained, subjects performed euhydrated and hypohydrated trials in a randomised crossover manner both before and after four exposures to the hypohydration stimulus. Euhydration or hypohydration (~ 2.4%) was produced by manipulating fluid intake in the 24 h before and during a 45-min steady-state run prior to a 5-km treadmill time trial. Hypohydrated performance before the four hypohydration exposures was significantly slower (–5.8%) than when euhydrated, but only 1.2% slower after familiarisation. Although there was no significant difference between hypohydrated and euhydrated trials after familiarisation, there was a strong trend (*p *= 0.064), with nine of the ten subjects running slower in the hypohydrated trial (compared to ten before familiarisation; Fig. 3). For all subjects, hypohydrated performance improved, while euhydrated performance did not change after familiarisation. Interestingly, responses for perception of effort during the 45-min steady-state run mirrored the performance effects observed.

Therefore, this study suggests that repeated familiarisation (on five occasions) with a hypohydration stimulus can attenuate, but not abolish, the negative performance effects of hypohydration, at least for running. This might go some way to explaining why well-trained endurance athletes (who will likely have years of exposure to hypohydration) might be able to finish and seemingly perform well in competitive events, despite sometimes substantial hypohydration at the end of the race [84–88, 92]. Clearly, further research is needed, but where maintenance of euhydration is not possible and athlete health and safety permits, perhaps strategic familiarisation with the anticipated hypohydration (method and magnitude) might be a prudent ergogenic strategy [95]. It is anticipated that for most athletes this would simply represent continuing normal weekly training, because, at least for endurance activities, training likely presents a similar or reduced opportunity to consume fluid (i.e. no drink stations, limited support/ability to carry fluids). However, in situations where fluid availability is low in competition and high in training, some ‘competition-specific fluid intake training’ might be beneficial.

It is also important to consider the results of the study of Fleming and James [39] in the context of the numerous other studies that have used uncomfortable hypohydration methods before testing performance capabilities. They suggest that the results of these previous studies, where uncomfortable and unfamiliar methods have been used to induce hypohydration, might exaggerate the negative performance consequences of hypohydration and, therefore, these studies should be carefully interpreted.

Furthermore, weight loss induced through dehydration has been theorised to possibly increase performance in activities where body mass must be carried [6]. However, Ebert et al. [31] reported that after a 2-hour cycling preload, time-to-exhaustion in uphill cycling (8% grade) was reduced with hypohydration of ~ 2.5% compared with euhydration, suggesting that hypohydration still impairs performance even when body mass must be carried. Moreover, a number of studies have reported that endurance running performance (i.e. where body mass is carried) is impaired by hypohydration [26, 32, 38], suggesting that, at least for unfamiliar/novel hypohydration, the associated mass loss is unlikely to be ergogenic. Whether this holds true for well-trained athletes used to experiencing the levels of hypohydration experienced during racing is unclear and clearly requires further work.

## Conclusions

### What We Know

This review mainly focuses on a small number of studies using contemporary methods to explore the effect of hypohydration on endurance performance. Whilst there are some inconsistencies among findings, we feel these can be reconciled, as discussed above, but the limited work in this area means it is difficult to draw strong conclusions about many relevant questions related to hypohydration and human performance. It is, however, possible to make some tentative conclusions at this time. Hopefully, future work in this area will continue to more robustly investigate the effects of hypohydration on endurance (and other types of) performance to allow better inferences to be made.

First, from the available evidence, it seems that hypohydration of > 2% body mass impairs endurance cycling performance, even when subjects are blinded to their hydration status. For performance to differ between euhydrated and hypohydrated trials, it appears they need to be differentiated by the main physiological effects of exercise-induced changes in hydration (i.e. differences in plasma volume and serum osmolality). Thirst perception also appears to play an important role, and whilst its independent effects on performance are unknown, it appears that the absence of thirst might be an important requirement for optimal performance when euhydrated. Therefore, to maximise performance, the prudent approach, in line with current guidelines [17], would be to prevent substantial hypohydration accruing during exercise. That said, what is ‘substantial’ is possibly difficult to define at an individual level. Whilst studies show that performance decrements are apparent when mean losses of 2% body mass occur, the large inter-individual variability in the deleterious nature of hypohydration (for example, − 1.5% to − 19.2% in our blinded hydration studies [41, 43]) makes it difficult to give a specific threshold. Some athletes may show performance impairments at low levels of hypohydration, whilst other athletes might be able to tolerate large levels of hypohydration. Therefore, a more individualised approach to athlete hydration should be considered, with particular attention paid to what fluid loss is practically and physiologically possible to replace in the context of a given athletic setting. It is important to note, that whilst not discussed here, fluid intake during exercise should not exceed fluid loss, a concept well reviewed elsewhere [6, 9]. In most scenarios, this recommendation is likely achieved by athletes simply drinking when thirsty, although some situations might require planned strategies to achieve this.

Second, when hypohydration is equivalent to ≥ 3% body mass, blinding does not appear to alter physiological, perceptual or performance responses. However, again, the performance effects in particular appear to hinge on whether all of the typical physiological (and possibly perceptual) effects are different between hypohydration and euhydration (i.e. differences in serum osmolality, plasma volume and possibly thirst). This is important, as it suggests that the results of previous studies achieving hypohydration of ≥ 3% body mass are unlikely to be exaggerated by any associated placebo/nocebo effects. Whilst hypohydration of 2–3% body mass clearly impairs performance, whether the results of previous studies where hypohydration was < 3% were inflated by placebo/nocebo effects is unknown. To be confident about the results obtained, future studies seeking to examine the performance effects of hypohydration should either use methods to blind subjects to their hydration status or achieve hypohydration of ~ 3%.

Third, it appears that some of the previously reported effects of hypohydration are possibly related to the discomfort associated with the dehydration methods used. Therefore, future studies are encouraged either not to use these often unrealistic and contrived methods to induce dehydration or to diligently familiarise subjects with the methods used (perhaps up to five times) to remove these effects. Similarly, in athletic settings where hypohydration is unavoidable and training nutritional strategies to better maintain hydration status are not possible, athletes may benefit from training under competition hydration conditions to familiarise themselves with changes in hydration they will likely experience. It is important to consider the health and safety of the athlete before considering these strategies, but this ‘training’ may attenuate negative performance effects associated with hypohydration.

### What We Do Not Know

To date, studies blinding subjects to hypohydration have only explored the effects on endurance performance, specifically cycling performance of men in warm/hot environments (30–35 °C). From previous unblinded work, this is perhaps the situation (exercise and environment) where one might expect hypohydration to compromise performance most [1, 4, 17]. Sweat losses are high in warm/hot environments, creating significant competition for blood flow demands between working muscle and skin. Additionally, because body mass is not being carried when cycling on a stationary bike, reductions in body mass with hypohydration are unlikely to mask performance impairments. Clearly, further work is needed to establish the effects of hypohydration in exercise/performance modalities other than cycling, as well as in cooler environments. Whilst it seems likely performance will be impaired in these settings, as thermoregulatory effects do not explain all the performance effects of hypohydration, until the research has been performed any conclusions would simply be speculation. Furthermore, because some of the effects of hypohydration are likely mediated by impairments in thermoregulation, the lack of appropriate facing air flow in some of these studies might amplify thermoregulatory differences between euhydration and hypohydration. Clearly, further work is required to explore these effects, but previous work has reported hypohydration still impairs performance when exercise is performed outdoors where facing airflow matches running/cycling speed [26, 32, 37, 38]. Additionally, understanding the performance effects of lower levels of hypohydration (~ 1%), where placebo/nocebo effects related to study blinding are more likely to influence performance, is also of interest/importance.

Finally, future investigations should also perform these studies in women. As with many areas of exercise physiology/sport nutrition, women have been largely understudied in relation to how hypohydration affects performance. The fact that research in women is limited is not, on its own, a rationale for research, unless there is a clear mechanistic basis for an expected differential response between sexes. Compared to men, women generally have lower relative and absolute body water, and core body temperature at rest and during exercise fluctuates over the menstrual cycle [96]. Both these effects may have implications for hydration performance research. Thus, women may not respond to hypohydration in a manner similar to men and responses might not be consistent over the menstrual cycle or possibly with different contraceptive use. Therefore, future work should seek to explore the potential moderating effect of sex in exercise-hydration studies, to better understand how women might be impacted by hypohydration.
